# Decreased Activity of Erythrocyte Catalase and Glutathione Peroxidase in Patients with Schizophrenia

**DOI:** 10.3390/medicina58101491

**Published:** 2022-10-19

**Authors:** Vladimir V. Djordjević, Jelena Kostić, Žilijeta Krivokapić, Dane Krtinić, Milica Ranković, Milan Petković, Vladan Ćosić

**Affiliations:** 1 Department of Psychiatry with Medical Psychology, Faculty of Medicine, University of Niš, 18000 Niš, Serbia; 2 College of Applied Health Sciences, 35230 Ćuprija, Serbia; 3 Department of Pharmacology, Faculty of Medicine, University of Niš, 18000 Niš, Serbia; 4 Center for Mental Health Protection, Clinical Center Niš, 18000 Niš, Serbia; 5 Department of Physiology, Faculty of Medicine, University of Niš, 18000 Niš, Serbia; 6 Center for Medical Biochemistry, Clinical Center Niš, 18000 Niš, Serbia

**Keywords:** catalase, glutathione peroxidase, schizo

## Abstract

*Background and Objectives:* Catalase and glutathione peroxidase (GPx) are important antioxidant enzymes that break down hydrogen peroxide (H_2_O_2_) in order to control its intracellular concentration, thus enabling its physiological role and preventing toxic effects. A lack or disruption of their function leads to the accumulation of hydrogen peroxide and the occurrence of oxidative stress. Accumulating studies have shown that the activities of key antioxidant enzymes are impaired in patients with schizophrenia. Since the published results are contradictory, and our previous studies found significantly higher erythrocyte superoxide dismutase (SOD) activity in patients with schizophrenia, the aim of this study was to determine the activity of enzymes that degrade hydrogen peroxide in the same group of patients, as well as to examine their dependence on clinical symptoms, therapy, and parameters associated with this disease. *Materials and* *Methods:* Catalase and GPx activities were determined in the erythrocytes of 68 inpatients with schizophrenia and 59 age- and gender-matched healthy controls. The clinical assessment of patients was performed by using the Positive and Negative Syndrome Scale (PANSS). The catalase activity was measured by the kinetic spectrophotometric method, while the GPx activity was determined by the commercially available Ransel test. *Results:* Erythrocyte catalase and GPx activities were significantly lower (*p* < 0.001 and *p* < 0.01, respectively) in subjects with schizophrenia than they were in healthy individuals. Lower catalase activity does not depend on heredity, disease onset, the number of episodes, or disease duration, while GPx activity showed significant changes in patients who had more than one episode and in those who had been suffering from the disease for over a year. Significantly lower catalase activity was noted in the PANSS(+/−) group in comparison with the PANSS(+) and PANSS(−) groups. The lowest catalase activity was found in subjects who were simultaneously treated with first- and second-generation antipsychotics; this was significantly lower than it was in those who received only one class of antipsychotics. *Conclusion:* These results indicate the presence of oxidative stress in the first years of clinically manifested schizophrenia and its dependence on the number of psychotic episodes, illness duration, predominant symptomatology, and antipsychotic medication.

## 1. Introduction

Antioxidative enzymes, including catalase and glutathione peroxidase (GPx), protect cells against reactive oxygen species (ROS)-mediated injury. Catalase is one of the most important antioxidative enzymes that catalyze the decomposition of hydrogen peroxide in two pathways, depending on its cellular concentrations. In the presence of high concentrations of H_2_O_2_, the catalytic pathway is favored—the two molecules of hydrogen peroxide are degraded into one molecule of oxygen and two molecules of water [1,2]. In the case of low concentrations of H_2_O_2_, the peroxidase pathway of catalase is activated with the oxidation of hydrogen donors such as phenols and alcohols [3]. This detoxification mechanism is very effective due to the wide distribution of catalase, especially if a large amount of hydrogen peroxide is generated [4]. This enzyme has a predominant role in the disposal of hydrogen peroxide within human erythrocytes [5].

GPx is a selenium-containing enzyme that is also involved in the efficient maintenance of hydrogen peroxide homeostasis and the prevention of oxidative damage. This enzyme breaks down hydrogen peroxide but also eliminates hydroperoxides and lipid peroxides through reduction using reduced glutathione (GSH). Furthermore, the GSH/glutathione reductase (GR) cooperates as a system in order to make the active and reduced form of the enzyme (GPx) available and ensure the reversibility of redox modifications derived from H_2_O_2_ [6]. Since catalase and GPx are localized in several cellular compartments such as peroxisomes, mitochondria, membranes, the cytosol, the endoplasmic reticulum, and the nucleus, these enzymes effectively maintain the optimal intracellular level of hydrogen peroxide that ranges from 1 nM to approximately 100 nM [7].

Normal aerobic metabolism in humans yields different types of ROS, including singlet oxygen, superoxide, hydrogen peroxide, and hydroxyl radicals. Among the major examples of ROS is hydrogen peroxide, a significant and relatively stable (under physiological conditions) oxygen non-radical species that is soluble in water and has a relatively long half-life. It may act as a weak oxidizing as well as a reducing agent. Although it is not very reactive, it is a progenitor of many other very reactive and toxic oxygen species. It has been observed that H_2_O_2_ may oxidatively modify enzymes by the oxidation of their essential thiol groups at the active sites [8], as well as the Fe-S clusters contained in dehydratases, the heme-containing enzymes, and the peroxidatic thiols [9]. In the presence of transition metal ions, H_2_O_2_ is transformed into a more reactive hydroxyl radical that is capable of altering cell lipids, proteins, and DNA [10]. However, under physiological conditions, it has a central role in homeostatic metabolism and plays a significant role in the regulation of cellular metabolism. Its main biological source is superoxide anion radicals. According to its chemical properties, superoxide is a weak acid, and it is generally a better reducing agent than an oxidizing one. Superoxide is relatively unreactive toward most biological molecules, and unlike hydrogen peroxide, it does not readily cross lipid membranes. In cells and tissues, this radical acts as a signaling molecule that regulates numerous biological processes, including apoptosis, aging, and senescence. Superoxide anion is mostly produced in mitochondria and by the Nox family of NADP (Nicotinamide-adenine dinucleotide phosphate) oxidase [11]. However, there are several potential sources in schizophrenia, such as the dysfunction of dopaminergic and glutamatergic neurotransmission systems, high concentrations of nitric oxide, and impaired energy generation in mitochondria [12,13]. The H_2_O_2_ produced in the cell diffuses across the cell membranes via aquaporin water channels [14] and activates ROS signaling with the oxidative modification of critical redox-sensitive Cys in signaling proteins such as transcriptional factors (TFs), mitogen-activated protein kinases (MAPKs), and protein Tyr phosphatases (PTPs) [15]. As a second messenger, H2O2 is a mediator of physiological processes such as cell differentiation and proliferation, migration, cellular metabolism, apoptosis, and immune response [7]. In order to fulfill those roles, its concentration is maintained within physiologically acceptable limits achieved by the activity of the antioxidant enzymes catalase and glutathione peroxidase. The increased production of hydrogen peroxide and/or the impaired activity of hydrogen peroxide-degrading enzymes lead to oxidative stress, which in turn leads to toxic responses and damages on the basis of cellular localization, concentration, and upstream/downstream interactions. Nowadays, it is clear that many human diseases, including schizophrenia [16,17,18], are associated with oxidative stress. The hypothesized role of oxidative stress in the pathophysiological mosaic of schizophrenia is supported by the mediation of oxidative stress in excitotoxic neuronal damage [19], its apoptotic trigger properties, and its possible role in dendritic and synaptic apoptosis [20]. Keeping in mind the importance of hydrogen peroxide control for the development of numerous physiological processes as well as the conflicting results related to enzymes that control its concentration, the aim of the study was to simultaneously determine the activity of both catalase and GPx in the erythrocytes of patients with schizophrenia.

## 2. Material and Methods

### 2.1. Subjects

This study included 68 inpatients of either gender (29 females and 39 males) with schizophrenia (mean age 32.7 ± 9.4 years), who were recruited from the Clinic for Psychiatry of Nis Clinical Center according to the following inclusion criteria: age group—18–65 years; confirmed diagnosis of schizophrenia using ICD-10 (The International Classification of Mental and Behavioral Disorders) and DSM-V (Diagnostic and Statistical Manual of Mental Disorders) diagnostic criteria; stable doses of oral antipsychotic drugs.

The exclusion criteria included the following: major medical illness associated with oxidative stress; any extent of drug or alcohol abuse/dependence; history of some other psychiatric or neurological disorder; subjects who received any immunomodulators or antioxidants. Following the clinical observation, patients’ medical and personal histories were recorded, and they underwent laboratory testing. Two independent psychiatrists participated in the diagnostic and psychopathological assessment by using the Positive and Negative Syndrome Scale (PANSS). According to the results of the PANSS, the patients were divided into three subgroups: one with a positive PANSS score predominance (PANSS(+)), one with a negative PANSS score predominance (PANSS(−)), and one with almost equally manifested positive and negative symptoms (PANSS(+/−)). Furthermore, patients were also divided into three subgroups according to the drug treatment: patients treated with haloperidol (first-generation antipsychotic—FGA), those treated with risperidone or olanzapine (second-generation antipsychotics—SGA), and patients simultaneously treated with FGA and SGA. Data related to age, gender, the onset and duration of the disease, the number of episodes, and heredity were obtained using medical documentation; auto-anamnestic and hetero-anamnestic data from patients’ family members were also encompassed in this study. At the same time, we also recruited 59 healthy subjects, (27 females and 32 males; mean age 30.9 ± 6.9 years) among the hospital medical staff, who represented the control group. The patients and controls were matched according to age, gender, marital status, living settings, and habits.

### 2.2. Methods

Following an overnight fast in the vacutainer test tubes with lithium heparin, venous blood samples were collected between 8 and 9 a.m. Plasma was separated by centrifugation at 3000 rpm for 10 min, the buffy coat was removed, and packed cells were washed three times with physiological saline. Erythrocyte suspension was used for the hemolysate preparation, aliquoted, and stored at −20 °C until the enzyme activities were to be measured.

Erythrocyte catalase activity was determined by the kinetic spectrophotometric method of Beutler [21] based on the hydrogen peroxide degradation. Previously prepared hemolysate samples were diluted 100 times before the measurement of catalase activity on the Beckman spectrophotometer DU-650 (Beckman, Beckman’s National Technical Laboratories, PA, USA). Catalase activity was expressed in units per gram of Hb × 10^4^.

The erythrocyte GPx activity in hemolyzate samples was determined using the commercially available Ransel test (Randox Laboratories Ltd., Crumlin, Co. Antrium, UK) on the Beckman autoanalyzer AU-680 in accordance with the instructions of the manufacturer. The reaction itself proceeds as follows: glutathione peroxidase catalyzes the oxidation of the reduced glutathione with cumene hydroperoxide. In the presence of glutathione reductase and NADPH, the oxidized glutathione is immediately translated into a reduced form with the simultaneous oxidation of NADPH to NADP+, which leads to a drop in absorbance at 340 nm. The enzyme activity was expressed in units per gram of Hb.

### 2.3. Statistical Analysis

The SigmaStat computer program was used for the analysis of the collected data. Statistical comparisons between various groups were tested by one-way ANOVA and by Student’s t-test. The results are presented as the mean ± SD. The correlation analysis was performed using Pearson’s coefficient. A value of *p* < 0.05 was used as the limit of statistical significance.

## 3. Results

We recruited 68 patients with chronic schizophrenia into our study—in the younger sub-group, we had already noted a significantly higher SOD activity. Since SOD generates H_2_O_2_, we tested hydrogen peroxide-degrading enzymes together with catalase and GPx in all our study participants. More detailed characteristics of the patients and healthy donors are presented in Table 1.

As can be seen in Table 2, the activity of both catalase (*p* < 0.001) and GPx (*p* < 0.01) was significantly lower in comparison to the controls. The patients’ catalase activity was 29% lower than the one noted in the control group, while the GPx activity was 12% lower in comparison to the control values. The GPx activity in male patients was found to be significantly lower (*p* < 0.01) than the one in female patients. Patients with negative hereditary predispositions had significantly lower GPx and catalase activities (*p* < 0.001; *p* < 0.0001) in comparison with the control values. Patients with positive hereditary predispositions only had catalase activity that was significantly lower (and *p* < 0.05) compared to the control group. Both patient groups, i.e., patients affected by the disease before the age of 24 and those with the disease onset occurring after the age of 24, had significantly lower catalase (*p* < 0.001 and 0.01, respectively) and GPx activities (*p* < 0.001 and *p* < 0.05, respectively) than the controls. More than one episode significantly decreased both GPx (*p* < 0.001) and catalase activity (*p* < 0.001), whilst one episode decreased only catalase (*p* < 0.001) in comparison with the control values. The catalase activity was lower in schizophrenia patients at the beginning of the disease (*p* < 0.01), while the GPx activity was lower in those who were suffering from it for over a year (*p* < 0.01). This finding is in accordance with the correlation coefficient being significantly negative between catalase activity and disease duration (r = −0.624, *p* < 0.01).

The summed-up results suggest the following: the activities of catalase and GPx in chronic schizophrenia patients are significantly lower than those in healthy individuals; the male patients have significantly lower GPx activity than the female ones; there is no association whatsoever between heredity and enzyme activities; the enzyme activities are reliant on the number of episodes and the duration of the disease.

Table 3 shows that both examined enzyme activities are significantly lower in the patients in comparison to the control individuals, regardless of the patient’s symptomatology. However, the PANSS(+/−) patient group had also significantly lower catalase activity in comparison with the PANSS(+) and PANSS(−) groups (*p* < 0.05, for both). Although the decrease in catalase activity was observed in all PANSS groups, the biggest decrease was noted in patients who simultaneously exhibited both negative and positive symptoms.

Catalase activity was significantly lower in all three types of therapy groups, while GPx was significantly lower in patients treated with SGA (*p* < 0.05) and in those treated with both FGA and SGA (*p* < 0.001) in comparison to the control values. The groups treated only with FGA or SGA had significantly higher catalase activity (*p* < 0.05) compared to the group treated simultaneously with both types of antipsychotics. These results show that FGA (haloperidol) did not have a significant effect on GPx activity, while both FGA and SGA induced the highest decrease in catalase activity when given simultaneously.

## 4. Discussion

The most important finding in this study is the significantly lower activity of catalase and GPx, which may demonstrate a reduced capacity of the patients’ antioxidative systems to degrade hydrogen peroxide, which can potentially lead to oxidative stress. For the time being, we do not have a reasonable explanation for the reported result. However, our previously published results showed significantly higher erythrocyte SOD activity in the same group of patients with schizophrenia [22], which could result in increased hydrogen peroxide production, which provides more substrates for catalase and GPx. It is well known that catalase is a “suicide” enzyme that is inhibited in the presence of non-physiologically higher peroxide concentration. Furthermore, peroxidases are unstable and are promptly inactivated by their substrate—hydrogen peroxide. Recently, in addition to the observed increased SOD activity, Li et al. [23] also found increased catalase activity and decreased levels of GPx at baseline in antipsychotic-naïve first-episode patients with schizophrenia. On the one hand, the significant increase in SOD activity might be a compensatory mechanism that is activated in the early stage of schizophrenic disease in order to protect against oxidative injury, whilst on the other hand, the increasing production of peroxide could deepen oxidant stress. Similar to our results, significantly lower catalase activity as well as lower GSH levels were observed in drug-naïve first-episode schizophrenic patients [24,25], but the Gpx activity was significantly higher in the patients than in the controls. If GPx is a GSH-dependent enzyme that may be inactivated by its own substrate, how is this discrepancy to be explained?

Abnormal antioxidant defense systems have been observed in patients’ peripheral blood, cerebrospinal fluid, and post-mortem brain tissues [26,27,28,29,30]. The majority of studies have noted abnormal activities of critical antioxidant enzymes [31,32], reduced levels of antioxidants [33,34,35], and increased levels of oxidative products in blood and cerebrospinal fluid [36,37,38]. The fact that the antioxidant N-acetylcysteine and omega-3 fatty acid significantly reduced the psychopathology in schizophrenia also confirmed the existence of oxidative stress in this disease [39,40,41], which is an occurrence that could be a good focus area for future pharmacotherapy [17]. A suggestion was recently made to establish the persistence of GSH-deficient subtypes and elucidate the role of the central antioxidant system in the disruption of brain structure and connectivity in the early stages of schizophrenia [42]. Research findings regarding untreated patients in the first episode of schizophrenia imply that oxidative stress is part of the pathophysiological mosaic of schizophrenia present before the onset of clinically manifested psychosis [43]. From a functional point of view, the two-way relationship between oxidative stress and neurotransmission is of such a nature that it places the relevance of oxidative stress in the context of neurochemical pathogenetic theories. For example, a state of hyperdopaminergia leads to a higher rate of dopamine auto-oxidation and ROS formation, followed by the disruption of glutamatergic transmission [44]. The possible role of ROS in dendritic and synaptic apoptosis [20] and the association of schizophrenia with oxidative stress through reduced levels of brain-derived neurotrophic factor (BDNF) [45,46] potentially includes oxidative stress in the course of neurostructural changes, clearly positioning it within the framework of neuroprogressive theories and not ruling out its role in pathological neurotransmission [13].

Although many published results prove the altered antioxidant enzyme activities in schizophrenia, they are still inconsistent and often conflicting, and they can be interpreted in different ways. While some studies find an increase in GPx activity in schizophrenia [47,48], in some studies, it is unchanged [49,50]; yet, in others, it is decreased [51]. When comparing the results related to schizophrenia patients and their healthy twin pairs, significantly lower activities of SOD, GPx, and catalase were found in patients, whereas the GPx activity was significantly higher in healthy twin pairs than that in the control group. A potential interpretation of these results is that the elevated GPx activity plays a protective role in healthy twin pairs [52]. However, the low activity of GPx, which is a selenoenzyme, may be a consequence of the following: a defect in a selenium-transport protein that is observed in schizophrenia, the oxidative/nitrosative alterations of the enzyme [53], or the suppressed GSH levels resulting from oxidative stress [54].

There is also a question mark over the way in which drugs affect antioxidant enzymes. In this study, we showed that FGA did not significantly change the GPx activity, while both FGA and SGA significantly decreased the catalase activity. The lowest catalase activity was found in patients treated with both type of medicaments, despite the evidence showing that therapy with FGA antipsychotics leads to the normalization of catalase activity [55]. These results indicate more pronounced oxidative stress in patients who received both types of antipsychotics. Clinical and animal studies showed that FGA might increase ROS production through the dopaminergic system by several mechanisms [56]. Some data indicate that second-generation antipsychotics may also affect GPx and catalase activity. There is evidence that paliperidone significantly decreased catalase activity, while the impact on GPx activity in rat brain tissues was insignificant [57]. The patients who had higher activities of SOD and catalase and lower GPx activity at baseline received risperidone monotherapy for 12 weeks, and the 12-week follow-up showed a GPx decrease after treatment in both responders and non-responders [23].

In contrast, Liu et al. [58] found higher GPx activity in female responders in comparison with non-responders after a 12-week treatment with risperidone, even though they did not observe gender differences in the antioxidative enzyme activities at baseline, as well as 3 months after treatment with risperidone. Therapy with atypical antipsychotics does not have a pronounced impact on the glutathione system but may improve the redox balance, show anti-inflammatory effects, decrease lipid peroxidation, and increase antioxidant levels [59].

When summarizing the results of some studies, an association of negative symptomatology with low GPx activity was observed [59]. In our study, low catalase activity was present in all PANSS subgroups, but the lowest activity was noted in patients expressing both negative and positive symptomatology. Heredity, disease onset, the number of psychotic episodes, and disease duration did not have any significant influence on catalase activity. However, multiple episodes and a longer disease duration were associated with significantly lower GPx activity. This is somewhat consistent with the understanding that persistent oxidative stress leads to a compensatory increase in some antioxidant enzyme activities and that the level of enzyme activity decreases when the disease becomes chronic [47]. This is also in agreement with our previously published results showing that patients younger than 34 years of age had significantly higher erythrocyte SOD activity in comparison with the older age group (over 34 years) [22].

There are at least four possible reasons that contribute to the contradictory results.

First, multiple clinical types of disease make this disease very heterogeneous. Second, heterogeneity is present in the same type of disease. Third, there are different stages of the patients’ disease in every type of disease. Fourth, tissue-specific changes may underlie pathophysiology [60].

The limitations of this study are as follows: (1) Schizophrenia is known to be a disease that exhibits a great heterogeneity of clinical symptoms. Our patient group consisted of inpatients with different clinical manifestations of schizophrenia, different durations of illness, different numbers of episodes, and different treatments. (2) There was no pre-testing washout period. (3) Markers of oxidative damage were not measured. (4) Patients’ medications taken prior to the upcoming treatment were not taken into account. (5) Smoking was not considered, although smoking is known to be highly prevalent in this patient population. (6) Obesity was also not taken into account despite the evidence [61] that it might increase oxidative stress in schizophrenia.

In conclusion, the study results confirm differences in erythrocyte activities of hydrogen peroxide-degrading enzymes between patients suffering from schizophrenia and healthy individuals. Significantly decreased catalase and GPx activity indicates a reduced capacity to degrade hydrogen peroxide. The changes are more perceptible in patients with more psychotic episodes, a longer disease duration, and almost equally represented positive and negative symptoms, as well as in those treated with both generation antipsychotics. However, better patient stratification and the simultaneous monitoring of oxidative stress biomarkers are required to understand the significance of the found antioxidant deficit.

## Figures and Tables

**Table 1:** Demographic and clinical data of the studied patients.

	Patients Controls
__	
Male/Female (n)	29/39 27/32
Age (years)	32.7 ± 9.4 30.9 ± 6.9
Heredity (+/−)	20
Age of disease manifestation:	
Before 20 years (n)	19
Between 20 and 24 years (n)	16
Between 25 and 29 years (n)	18
Between 30 and 34 years (n)	15
Duration of psychiatric disease (years):	
<1	8
1–3	16
3–5	18
>5	26
Number of episodes (one/more than one, n)	26/42
PANSS positive scores predominant (>3) (n)	24.5 ± 6.9 (27)
PANSS negative scores predominant (<−8) (n)	24.7 ± 9.1 (19)
PANSS positive and negative scores almost equally expressed (>−8 < 3) (n)	(22)
PANSS general psychopathology	48.9 ± 9.1
PANSS total score	98.1 ± 25.1
FGA (haloperidol-treated, n)	22
SGA (risperidone- or olanzapine-treated, n) FGA- and SGA-treated, n	20 26

**Table 2:** Erythrocyte catalase and GPx activities in schizophrenia patients in relation to demographic and clinical characteristics.

	Patients		Controls	
	Cat	GPx	Cat	GPx
Total	9.1 ± 3.8 **	40.8 ± 8.9 *	12.7 ± 3.6	46.4 ± 8.3
Male	6.2 ± 1.1	35.5 ± 8.2 ^A^	14.5 ± 3.3 ^A^	47.2 ± 9.1
Female	6.6 ± 1.6	43.2 ± 6.6	11.5 ± 3.1	46.9 ± 6.7
Heredity (+)	10.5 ± 4.6 ^B^	43.8 ± 8.4	-	-
Heredity (−)	8.6 ± 3.4 ^C^	39.7 ± 7.9 **	-	-
Age of onset (before 24 y)	8.6 ± 3.89 **	39.6 ± 6.8 **	-	-
Age of onset (after 24 y)	9.96 ± 3.98 *	41.2 ± 11.4 ^B^	-	-
Number of episodes (one)	8.5 ± 4.1 **	42.7 ± 8.9	-	-
Number of episodes (more than one)	9.8 ± 3.8 **	39.0 ± 9.5 **	-	-
Disease duration (to one year)	9.1 ± 2.7 *	45.1 ± 10.6		-
Disease duration (less than 5 years)	9.5 ± 4.4 *	38.9 ± 9.7 *		-
Disease duration (more than 5 years)	9.1 ± 8.1 **	39.4 ± 8.1 *		-

*—*p* < 0.01 vs. Control; **—*p* < 0.001 vs. Control; A—*p* < 0.01 vs. Female; B—*p* < 0.05 vs. Control;. C—*p* < 0.0001 vs. Control.

**Table 3:** Erythrocyte catalase and GPx activities in patients with schizophrenia related to the predominant symptomatology and drug treatment.

	Patients	Controls		
	Catalase (U/gHb × 10^4^ )	GPx (U/gHb)	Catalase ((U/gHb × 10^4^)	GPx (U/gHb)
PANSS (+)	10.4 ± 4.2 ^A^	42.1 ± 8.1 ^A^	12.7 ± 3.6	46.4 ± 8.3
PANSS (−)	10.1 ± 3.6 ^A^	38.2 ± 8.3 ^B^	-	-
PANSS (+/−)	6.9 ± 2.9 *,**, ^B^	38.9 ± 10.4 ^A^	-	-
FGA	10.4 ± 4.6 ^¶^,^A^	42.9 ± 8.9	-	-
SGA	10.1 ± 3.7 ^¶^,^A^	39.0 ± 10.8 ^A^	-	-
FGA and SGA	7.9 ± 2.7 ^B^	39.2 ± 8.7 ^B^	-	-

A—*p* < 0.05 vs. control; B—*p* < 0.001 vs. control; *—*p* < 0.05 vs. PANSS (+); **—*p* < 0.05 vs. PANSS (−); ¶—*p* < 0.05 vs. FGA and SGA.

## Data Availability

The data that support the findings of this study are available on request from the corresponding author. The data are not publicly available due to privacy and ethical restrictions.
